# A manual-based vocational rehabilitation program for patients with an acquired brain injury: study protocol of a pragmatic randomized controlled trial (RCT)

**DOI:** 10.1186/s13063-017-2115-0

**Published:** 2017-08-10

**Authors:** Louise K. Hoeffding, Maria Haahr Nielsen, Morten A. Rasmussen, Anne Norup, Juan Carlos Arango-Lasprilla, Ulrikka K. Kjær, Kristoffer Sølvsten Burgdorf, Trine Schow

**Affiliations:** 1 Research and development, Brain Injury Center BOMI, Maglegaardsvej 15, 4000 Roskilde, Denmark; 2 Institute of Biological Psychiatry, Mental Health Centre Sct. Hans, Copenhagen University Hospital, Roskilde, Denmark; 3 Department of Clinical Immunology, the Blood Bank, Rigshospitalet, University Hospital of Copenhagen, Copenhagen, Denmark; 4 Faculty of Science, Chemometrics and Analytical Technology, University of Copenhagen, Copenhagen, Denmark; 5 Copenhagen Prospective Studies on Asthma in Childhood, Faculty of Health and Medical Sciences, University of Copenhagen & Danish Pediatric Asthma Center, Gentofte Hospital, University of Copenhagen, Copenhagen, Denmark; 6 National Study on Young Brain Injury Survivors, Department of Neurology, Rigshospitalet-Copenhagen University Hospital, Copenhagen, Denmark; 7 BioCruces Health Research Institute, Cruces University Hospital, Barakaldo, Spain; 8 IKERBASQUE. Basque Foundation for Science, Bilbao, Spain; 9 Kjaer Management, 3520 Farum, Denmark

**Keywords:** Traumatic brain injury, Vocational rehabilitation, Caregiver intervention, Return to work, Randomized controlled trials, Study protocol

## Abstract

**Background:**

An acquired brain injury (ABI) is a complex injury often followed by a broad range of cognitive, physical, emotional, and behavioral disabilities. Because of these disabilities, vocational rehabilitation (VR) is a challenging task, however, of great importance, since approximately 75% of the patients with ABI are of working age. Thus, standardized clinically effective and cost-effective methodologies regarding VR for patients with ABI are highly needed. Therefore, the aim of this study is to evaluate the effect of an individually targeted manual-based VR for patient with ABI compared to conventional VR (usual care).

**Methods:**

This study is an interventional, two-arm, six-month follow-up, cluster randomized controlled trial involving four municipalities in the Zealand Region and the Capital Region of Denmark. A total of 84 patients with ABI evenly distributed across four municipalities will be included in the study. The patients will randomly be allocated in a 1:1 ratio to the VR intervention provided by a specialized Brain Injury Centre or the conventional VR provided by the municipalities (usual care). The six- to nine-month intervention will consist of individual and group therapies as well as a work placement program including supported employment. Furthermore, the intervention will include a family intervention program followed up by support to one individual family caregiver. The primary outcomes are increased work or study rate at six-month follow-up. Moreover, a budget impact analysis and possibly a cost-utility analysis of the intervention will be performed.

**Discussion:**

This study consists of a comprehensive multidiciplinary VR intervention involving several parties such as the municipalities, a specialized rehabilitation team, and patients’ own family caregivers. If this intervention is proven successful when compared to the conventional VR, it will provide evidence for a manual-based individualized holistic approach in returning to work after an ABI. Furthermore, the study will contribute with novel knowledge regarding feasibility and clinical effectiveness of the VR intervention relevant to clinicians, researchers, and policymakers.

**Trial registration:**

ClinicalTrials.gov, NCT03086031. Registered on 21 March 2017.

**Electronic supplementary material:**

The online version of this article (doi:10.1186/s13063-017-2115-0) contains supplementary material, which is available to authorized users.

## Background

An acquired brain injury (ABI) is a complex injury often followed by a broad range of cognitive, physical, emotional, and behavioral disabilities [1, 2]. Because of these disabilities, most patients with ABI find it challenging to remain in or return to work post injury [3–5], even though approximately 75% of these patients are of working age [6].

Vocational rehabilitation (VR) is often a prolonged process, leading to high cost to the healthcare system and society [7]. Barriers to VR success include the severity of the ABI, age at injury, income, and education pre injury, but also challenges directly related to the workplace [1, 8, 9]. Often, the employer has little or no knowledge about the consequences of an ABI and there is no direct communication between the brain injury specialist, the employer, and the employees regarding the challenges and needs in relation to work-related tasks.

Even though returning to work has proven important for long-term ABI survival and increased levels of quality of life [10], standardized evidence-based VR programs targeting patents with ABI have been lacking [11–13]. One very interesting feasibility randomized controlled trial (RCT) protocol has been published regarding brain injury and return to work; however, this protocol involved patients with a traumatic brain injury exclusively [14]. It has previously been shown that support from specialized VR teams help patients with ABI returning to work post injury [1, 2]. However, only few studies have examined the long-term effect of different types of VR interventions [5, 13, 15–20]. Such studies have mainly focused on VR programs where multiple unstandardized components interact in time, which challenge the reproducibility and knowledge of the active modules involved. Moreover, the previous VR interventions have been performed similarly to all patients even though ABI is a broad diagnostic category with various degrees and characteristics. Thus, VR interventions that are tailored and adjusted to each patient might be proven more efficient and effective in enhancing returning to work.

The bidirectional relationship between the wellbeing of the patient and the family is well documented [21–29]; consequently, active participation from family caregivers is likely to show a positive effect on the reintegration of the patient in a work setting. Thus, family caregivers could be a valuable resource both in parallel to the professional support and to ensure future consolidation and anchoring of the VR.

In this study, we hypothesize that patients with ABI need a coordinated effort across multiple disciplines and sectors with specialized knowledge (such as municipalities, family caregivers, and rehabilitation teams) in order to get a coherent VR program. Therefore, the aim of this study is to develop an individually targeted manual-based VR program based on thoroughly described modules that inform clinicians, researchers, and policymakers about effectiveness and costs of the VR intervention.

## Methods/Design

### Trial design and hypotheses

**Fig. 1:**
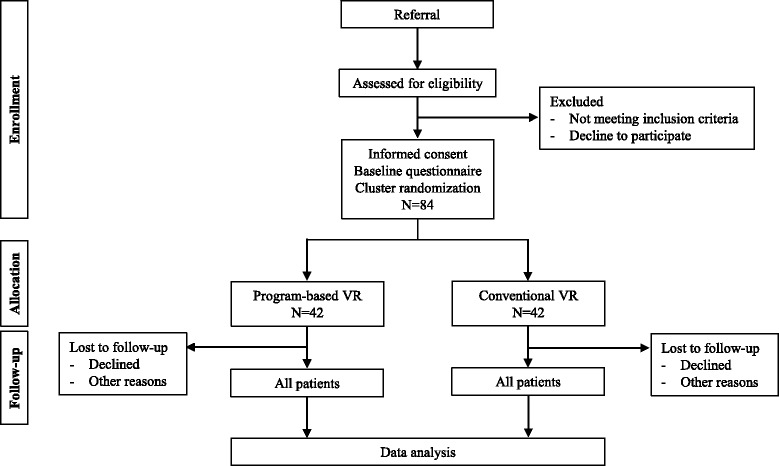
A *flow chart* of the study

An interventional, two-arm, six-month follow-up, cluster RCT (Fig. 1) including 84 participants with an ABI allocated randomly (allocation ratio 1:1) to the VR intervention and control group that consists of a conventional VR program (usual care).

The main objective of the study is to develop an individually targeted manual-based VR program and determine its efficacy for patients with ABI. The first hypothesis of the study is that more participants allocated to the VR intervention group will have a significant higher employment or study rate (measured in hours) at six-month follow-up when compared to participants receiving the conventional VR program provided by the municipalities (control group). The second hypothesis is that health-related quality of life (HRQoL) and disability among the participants and HRQoL and the caregiver burden among the caregivers are significantly improved at the end of the VR intervention and at six-month follow-up when compared to the conventional VR program. Finally, a budget impact and possibly a cost-utility analysis will be performed since we hypothesize that it will be an economically sound decision for the municipalities to invest in a specialized VR when compared to the conventional VR program provided by the municipalities. Such an analysis is based on an overall assessment of the health gain, both in terms of clinical effects and participant perceived utilities, and the related expenditures and costs.

The duration of the study is three years and the assessments will take place at baseline and at the end of the intervention (approximately after 6–9 months). In order to determine any long-term effect of the intervention, follow-up assessment is planned after six months.

**Fig. 2:**
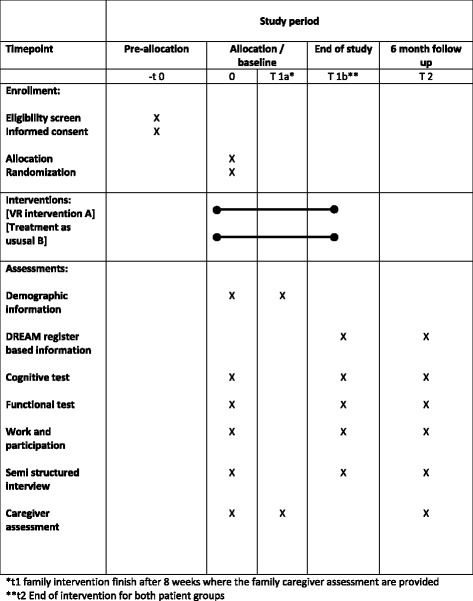
Content for the schedule of enrollment, interventions, and assessments

The trial is designed and reported according to the SPIRIT (Standard Protocol Items: Recommendations for Interventional Trials) Statement [30] (see Additional files 1 and 2 and Fig. 2).

### Patient recruitment and eligibility

The 84 patients included in the study will be recruited from several municipalities in the Zealand Region and Capital Region of Denmark in the period between August 2017 and August 2018. Before the beginning of the study the case managers at the municipalities will receive study training to be able to inform about the study and apply the inclusion criteria to each identified patient. Patients are eligible if they: (1) are aged 16–60 years; (2) suffer from an ABI; (3) acquired the injury within the last 3–24 months; (4) are employed or enrolled in education prior to the injury; (5) are currently employed or enrolled in education up to a maximum of 15 h/week or taking sick leave/unemployed; (6) have the motivation to participate in VR; (7) are able to participate in group sessions; (8) are able to speak and understand Danish; and particularly for the family intervention, (9) have at least two family caregivers willing to participate in the study. Individuals are excluded from the study if they: (1) have any other somatic or psychiatric illnesses before the ABI that make it impossible to complete the intervention; (2) suffer from substance abuse or severe apasia; (3) have a progressive brain injury; (4) are pregnant; and particularly for the family intervention, (5) have a mini mental state examination score < 23. All individuals that meet the inclusion criteria will be invited to enroll in the study and will be asked for consent to participate.

All patients in the intervention group will not receive usual care from the municipalities. However, they will not be stopped from participating in leisure activities or private practice treatment if they prefer this during the intervention period.

### Randomization

To avoid confounding between the VR intervention and the control group due to random probability, the number of cases and controls within each of the enrolled municipalities is set to be the same (+1/–1). Due to the prospective nature of the inclusion, which practically means that the number of patients included within each municipality is not known a priori, the randomization is performed by randomly assigning the first patient within each municipality to either the VR intervention or the conventional VR program. The following patient (within each municipality) is then assigned consecutive to the other group and so forth. This randomization scheme ensures equal distribution of cases and controls, both within each municipality as well as in time, avoiding confusion of these two factors. Controlling the distribution over time is central in such a study where some variation over the trial period due to protocol familiarity of the rehabilitation team at the Brain Injury Centre may be expected.

### Blinding

Because of the nature of the study, it will be clear to most participants whether they are allocated to the VR intervention or the control group. Thus, it is not possible to blind either the participants or the rehabilitation team, but they will be asked not to disclose any details about the allocation or the content of the VR. The two independent research assistants will be trained to administer the assessments and will be blinded to the allocation when entering data into the database. The researchers and the statistician performing the data analysis are blind to the treatment arm in which the participants are allocated. The rehabilitation team and the research assistant will only be involved in the data collection and will therefore not participate in the data analyses.

### A description of the manual-based VR interventions

This manual-based VR study comprises the following three phases:

1. Initial assessment: Within two weeks after the referral to the study, each patient will meet with the case manager in the municipality (brain injury coordinator). Here, a more thorough interview will ensure that the inclusion criteria are met and, in addition, asses the patient’s readiness for VR. Moreover, the participant will be fully informed about the content of the study, how participation is voluntary, and how they are free to withdraw at any time during the study. If written informed consent is obtained, each patient is assigned a unique trial ID number and baseline questionnaires will be filled out together with the following socio-demographic, employment, and clinical data: gender; date of birth; residence; civil status; nationality; income; pre-injury employment and education status; pre-injury occupation status; length of education; time since the brain injury; type and location of the brain injury; number of days hospitalized; the date of discharge; clinical diagnoses or any relevant medical events; and alcohol consumption or abuse. Participants will then be randomly assigned to either the VR intervention or the conventional VR. At last, each patient included in the VR intervention group will be asked to choose at least two family members to participate in the family intervention and one family caregiver which will participate actively in the individual sessions and serve as future support and resource facilitator to assist them in returning to work. This initial assessment will take approximately 50 min and the patient will have the opportunity to ask questions throughout the entire assessment. To exclude recruitment bias, patients that meet the eligibility criteria but refuse to participate in the study will be asked a few questions about their refusal. Hereafter, the patient will be contacted by external testers and baseline testing will be completed.
2. The VR intervention program: The VR intervention lasts for a total of 6–9 months and consists of, first, six different modules (approximately three months) that are individually planned based on the patient needs and goals and, second, a work placement program (approximately 3–6 months). To ensure a uniform procedure of each of the different modules, comprehensive “standard operating procedures” (SOPs) will be developed by the rehabilitation team at the Brain Injury Centre before the beginning of the VR intervention. All members of the rehabilitation team (which includes six neuropsychologists, eight occupational therapists, and four physiotherapists) are already experienced in the VR intervention program and exceedingly specialized in ABI rehabilitation. They will go through a one-day workshop to be thoroughly introduced to the specific study protocol and the SOPs as recommended by Holmes et al. [31]. The six modules will be grouped into: (1) individual therapies, which includes neuropsychological sessions (10 h), balance between work and everyday life (20 h), and job matching (10 h); (2) grouped-based therapies, which includes psycho education (18 h), mindfulness (15 h), and physiotherapy training (15 h); (c) a manualized family intervention program (eight sessions of 90 min) [32] and an individual caregiver coaching, supporting the caregiver in assisting the patient using the different strategies and tools learned in the intervention in everyday life and at the workplace (12 h); (4) an individual work placement program including work practice (3–6 months), supported employment where the rehabilitation team is at the work place (30 h), and the development of a post-rehabilitation plan (4 h). The modules are planned based on the participant’s needs and goals thus, the length, duration, structure, and composition of the program will vary from patient to patient. The individual sessions and group sessions are delivered either in parallel or in serial depending on the patient.
  Group sessions will take place at the Brain Injury Centre BOMI and the individual sessions will most often take place in the patient’s home. The individual work placement intervention will take place at relevant workplaces, the patient’s current workplace, or at a new workplace found by the municipality or the Brain Injury Centre BOMI, depending on the individual case.
3. Post-trial evaluation: An evaluation of the VR intervention will be performed by a self-developed semi-structured interview of each patient. Here, questions related to the recruitment procedure, the modules of the study, the timing of modules, and the coaching by the rehabilitation team will be collected. Furthermore, all case managers at the municipalities that referred participants to the study will be asked about their experiences and level of satisfaction.

### The conventional VR provided by the municipalities (usual care)

Individuals allocated to the control group will receive the conventional VR program provided by the municipalities over the same period of time. Thus, the participants in the control group will receive VR support by the local municipal authority that may vary in content and intensity. As for the intervention group, each individual in the control group will select a family caregiver that will go through the same questionnaires as the caregivers in the VR intervention group regarding HRQoL and functional level.

Furthermore, the case manager at the municipalities is obliged to: (1) refer patients to the study; (2) hand out the baseline questionnaires to the participants and their family caregivers; and (3) complete a questionnaire about each participant at the beginning and end of the study, and again at the six-month follow-up.

An evaluation of the conventional VR provided by the municipalities will be performed by the rehabilitation team using a self-developed semi-structured interview of each patient.

### Outcome measures

Primary and secondary outcomes are outlined in Table 1 and are measured at baseline, after the end of the intervention, and at the six-month follow-up. In addition, the family caregivers will be evaluated (Table 1).

**Table 1:** Primary and secondary outcomes and outcomes related to family caregivers

Outcome	Assessment	Test tool	Assessment time:		
			Baseline	End	6-month FU
Primary outcome	Hours at work/study per week	Register-based or by interviews	**X**	**X**	**X**
Secondary outcomes	Proficiency on functional task	The Mayo-Portland Adaptability Inventory (MPAI-4) (participation index – rating will be done separately by the patient, relatives, and staff)	**X**	**X**	**X**
	Functional status	Glasgow Outcome Scale Extended (GOSE)	**X**	**X**	**X**
	Health-related quality of life	EuroQol Five Dimensions Questionnaire EQ-5D-3 L	**X**	**X**	**X**
	Anxiety and depression	Hospital Anxiety and Depression Scale (HADS)	**X**	**X**	**X**
	Fatigue	Dutch Multifactor Fatigue Scale (DMFS)	**X**	**X**	**X**
	Physical functioning	Timed Up and Go (dual task) (TUG)	**X**	**X**	**X**
	Self-perceived burden to others	Self-Perceived Burden Scale (SPB)	**X**	**X**	**X**
	Socio-demographic, employment, and clinical data	Structured interviews	**X**	**X**	**X**
	Working ability	Work Ability Index™ (version 2007)	**X**	**X**	**X**
	Assessment of intelligence	Wechsler Adult Intelligence Scale (subtest)	**X**	**X**	**X**
	Executive functioning	TRAIL Making Test A and B	**X**		
	Cognitive functioning	Paced Auditory Serial Addition Task (PASAT)	**X**	**X**	**X**
	Working memory	Connors Continuous Performance Test	**X**	**X**	**X**
Outcome family caregivers	Quality of life	EuroQol Five Dimensions Questionnaire EQ-5D-3L, SF-12	**X**	**X**	**X**
	The adaptability and cohesion dimensions in family interactions	Family Adaptability and Cohesion Scale (FACES-IV)	**X**	**X**	**X**
	Problem-solving ability	Problem Solving Inventory (PSI)	**X**	**X**	**X**
	Assess perceived burden among caregivers	Caregiver Burden Scale (CBS)	**X**	**X**	**X**
		Hospital Anxiety and Depression Scale	**X**	**X**	**X**

### Economic evaluation of the intervention

The economic consequences of the ABI will be evaluated using a two-stage approach. First, a budget impact analysis of the financial streams related to ABI for the two compared interventions will be performed to examine the affordability of the interventions. Second, the health gains will be quantified by the standardized questionnaire EQ-5D-3 L (health profiles and self-perceived health status). If relevant and feasible, a cost-utility analysis (quality adjusted life-years [QALYs]) from a societal perspective will be performed, including a cost-effectiveness acceptability curve to show the probability that the intervention will be cost-effective for a range of maximum prices that the municipalities will be willing to pay for an extra QALY.

Data on financial streams and costs are collected from the patients when discharged from hospital to the end of the follow-up period of the study by a self-developed questionnaire to the participating municipalities. Data collection will be based on routinely collected data, which can be retrieved from financial and administrative systems, in order to get an overall cost estimate on consumption of handicap compensating products and services (if the consumption of such services deviates significantly for the two types of interventions, i.e. marginal cost perspective). Non-priced usage of welfare services will be based on time usage and converted into monetary value by standard prices or local costs. Long-term effects on employment will be forecasted based on the observed employment effects during the follow-up period. Age-specific assumptions regarding employment will be set in order to calculate the averaged long-term effects.

In order to calculate the present value of the future gains of the interventions, a discount rate of 4% will be applied. Statistical uncertainty on the results will be calculated, in addition, to sensitivity analysis on critical assumptions (difference-in-difference analysis).

### Data collection and management

Data will be stored in a secure password-protected electronic database hosted by the Brain Injury Centre BOMI following Danish legislation. Over a period of three years, 84 individuals with ABI will be included in the study and assigned a unique trial ID number. Data will be collected prior to the intervention (baseline), at the end of the intervention, and at the six-month follow-up by various questionnaires (Fig. 1 and Table 1). All assessments will take place at the local rehabilitation center and will be done by two to three research assistants blinded to the intervention. Each assistant will receive thorough training in the assessment and will continue to be supervised throughout the study period. Data will be entered directly into a survey and exported into the database to ensure quality of the data. Access to data is restricted to the principle investigator and authorized researchers reported to the Danish Data Protection Agency. Furthermore, the data will be published in peer-reviewed scientific journals and the results will also be presented at national and international conferences.

### Ethical issues

The study has been reported to the Danish Data Protection Agency (registration no. 2016-41-4950) and is registered with the Clinical Trials Gov identifier no. NCT03086031. The Regional Ethical Committee of Copenhagen, Denmark, has stated that the study does not need their approval (komitélovens §§1 and 2). The study will be carried out according to the local legal and regulatory requirements and data will be handled according to the guidelines given by the Danish Personal Data Protection Agency.

All participants must give written and oral informed consent to participate in the study, including their consent to publish the results. If any participants are under the age of 18 years, they will provide a verbal acceptance while their parents will complete the written consent.

We expect a very low risk of adverse effects. However, increased fatigue in the intervention group could occur since a more intense program is followed and the test situation might be exhausting to some patients. Furthermore, VR could for some participants be perceived as stressful and inclusion of the family caregivers could result in a higher risk for intra-family conflicts. However, the sessions in the family intervention program includes psychoeducation in how to recognize, handle, and cope with situations that might escalate emotionally. All adverse effects will be reported and participants have the possibility to contact the rehabilitation team by telephone or email throughout the study.

### Sample size and power

**Fig. 3:**
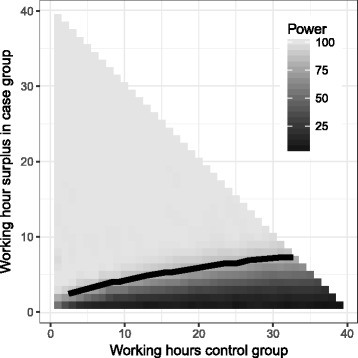
The *x-axis* depicts the average hours at work/study per week for the conventional VR treatment and the *y-axis* the average surplus obtained from the VR intervention. The *colors* indicate the study power at level alpha 0.05 under these expectations for a study with 42 + 42 = 84 participants. The *black line* indicates the combinations obtaining a power of 0.8

The primary endpoint “hours at work/study per week” is assumed to follow a Poisson distribution with an overdispersion of 4. Under this assumption, the relation between average hours at work for the conventional VR treatment, the expected surplus of hours by the VR intervention and the study power is calculated for a study with n = 42 + 42 = 84 participants. Figure 3 shows this relation where, for instance, a study with 10 h of work for the conventional VR treatment and 14 h of work for the VR intervention leads to a study power of 0.8 at level alpha = 0.05.

### Statistical analysis

Data analysis will be conducted using the statistical program R version 3.2.2. Baseline characteristics of the participants will be reported to ensure homogeneity between the VR intervention and the control group (reported as mean and standard deviation for continuous variables and proportions for categorical variables). Differences between baseline characteristics for continuous variables will be tested by an independent samples *t*-test or a Mann–Whitney U test dependent on the distribution of the data. Categorical variables will be analyzed by a Chi-square test. If any significant differences between the VR intervention and the control group are found, these will be adjusted for in the downstream analyses.

The differences between the VR intervention and the control group for the primary and secondary outcomes will be analyzed using repeated measurements statistical tools and multilevel analysis, including possible confounders (e.g. age, gender, ABI severity, socioeconomic status pre-injury, education, or recruitment site). *P* values and 95% confidence intervals will be provided and *P* < 0.05 will be considered statistical significant.

### Trial status

The trial is expected to start recruiting the first participants in August 2017. Follow-up assessments, including data analysis, are expected to be completed by December 2019.

## Discussion

The objective of this study is to develop an individually targeted manual-based VR program for patients with an ABI and evaluate its effectiveness measured as hours in employment/study and costs when compared to conventional VR provided by the municipalities.

To our knowledge, this is one of the first studies to take a holistic approach and formalize the collaboration between municipalities, a specialized rehabilitation team, and family caregivers in order to improve VR for patients with ABI. The results of this study will provide several innovative contributions to the existing literature regarding the feasibility and effectiveness of this VR intervention important for the allocation of resources in future VR programs.

An important element in this VR program is the individualized approach, aiming to support the participant in the work/everyday life balance, cognitive, and functional problems as well as providing individual support for gaining and sustaining employment. In Denmark, the municipalities have the responsibility to offer rehabilitation and therefore an important issue in this study is to focus on the cooperation with the municipalities. The different elements of this VR program will be described in separate manual-based procedures increasing the knowledge of the different “ingredients” used to the different participants. Thus, the positive effect of this program should provide measurable progress in employment status and participation, level of fatigue, functional disability, and HRQoL. However, the study will not be able to determine which element or elements in the VR program are the most effective or if some could be omitted or increased in intensity. Instead, this study will provide knowledge about how the VR intervention differentiates from the program in the municipality, since the elements (modules) are described and the effect and utility costs are evaluated.

The study gains its strength from the participation of various municipalities spread across the Zealand Region and the Capital Region of Denmark that will ensure that different types of ABI will be represented in the study. An additional strength is the inclusion of family caregivers in the VR intervention, which facilitate that the effects will be implemented and anchored after the VR intervention ended that could otherwise be lost. Furthermore, the family intervention program addresses the family function as a whole, which more or less is an overlooked issue in Danish rehabilitation.

The study is limited by a number of factors. First, the study will not include patients that for some reasons are unwilling to participate in the study but fulfill the inclusion criteria. However, when possible, the reason for refusal and some baseline questions will be obtained and included in the analysis in order to avoid recruitment biases. Second, both the participants and the rehabilitation team will not be blinded to the allocation, but the cluster randomization and blinding of the researchers should ensure that contamination does not occur. Third, the control intervention may most likely vary across the municipalities that participate in the study and may also vary nationwide, affecting the social benefits and vocational terms.

## Additional files

Additional file 1: SPIRIT Checklist. (DOC 123 kb)

Additional file 2: Informed consent. (DOC 122 kb)

## Abbreviations

ABI: Acquired brain injury
CBS: Caregiver Burden Scale
DMFS: Dutch Multifactor Fatigue Scale
EQ-5D-3L: EuroQol five dimensions questionnaire
FACES-IV: Family Adaptability and Cohesion Scale
GOSE: Glasgow Outcome Scale Extended
HADS: Hospital Anxiety and Depression Scale
HRQoL: Health-related quality of life
MPAI-4: The Mayo-Portland Adaptability Inventory
PSI: Problem Solving Inventory
QALY: Quality adjusted life-years
SF-12: 12-Item Short Form Health survey
SOP: Standardized operating procedure
SPB: Self-Perceived Burden Scale
TUG: Timed Up and Go
VR: Vocational rehabilitation
