# Erratum to: A manual-based vocational rehabilitation program for patients with an acquired brain injury: study protocol of a pragmatic randomized controlled trial (RCT)

**DOI:** 10.1186/s13063-017-2146-6

**Published:** 2017-09-06

**Authors:** Louise K. Hoeffding, Maria Haahr Nielsen, Morten A. Rasmussen, Anne Norup, Juan Carlos Arango-Lasprilla, Ulrikka K. Kjær, Kristoffer Sølvsten Burgdorf, Kirsten Jensen Quas, Trine Schow

**Affiliations:** 1 Research and development, Brain Injury Center BOMI, Maglegaardsvej 15, 4000 Roskilde, Denmark; 2 Institute of Biological Psychiatry, Mental Health Centre Sct. Hans, Copenhagen University Hospital, Roskilde, Denmark; 3 Department of Clinical Immunology, the Blood Bank, Rigshospitalet, University Hospital of Copenhagen, Copenhagen, Denmark; 4 Faculty of Science, Chemometrics and Analytical Technology, University of Copenhagen, Copenhagen, Denmark; 5 Copenhagen Prospective Studies on Asthma in Childhood, Faculty of Health and Medical Sciences, University of Copenhagen & Danish Pediatric Asthma Center, Gentofte Hospital, University of Copenhagen, Copenhagen, Denmark; 6 National Study on Young Brain Injury Survivors, Department of Neurology, Rigshospitalet-Copenhagen University Hospital, Copenhagen, Denmark; 7 BioCruces Health Research Institute, Cruces University Hospital, Barakaldo, Spain; 8 IKERBASQUE. Basque Foundation for Science, Bilbao, Spain; 9 Kjaer Management, 3520 Farum, Denmark; 10 Neuropsychologist, Head of Research and Development at Brain Injury Center BOMI, Roskilde, Denmark

## Erratum

The original publication [1] misses one author. The original article has been updated to rectify this error. All details about the missing author can be found below:

Kirsten Jensen Quas, Neuropsychologist, Head of Research and Development at Brain Injury Center BOMI, Roskilde, Denmark.
